# Integrated Laser Sensor (ILS) for Remote Surface Analysis: Application for Detecting Explosives in Fingerprints

**DOI:** 10.3390/s19194269

**Published:** 2019-10-01

**Authors:** Violeta Lazic, Antonio Palucci, Luigi De Dominicis, Marcello Nuvoli, Marco Pistilli, Ivano Menicucci, Francesco Colao, Salvatore Almaviva

**Affiliations:** Energy and Sustainable Economic Development (ENEA), Italian National Agency for New Technologies, FSN-TECFIS-DIM, Via E. Fermi 45, 00044 Frascati (RM), Italy; antonio.palucci@enea.it (A.P.); luigi.dedominicis@enea.it (L.D.D.); marcello.nuvoli@enea.it (M.N.); marco.pistilli@enea.it (M.P.); ivano.menicucci@enea.it (I.M.); francesco.colao@enea.it (F.C.); salvatore.almaviva@enea.it (S.A.)

**Keywords:** LIBS, Raman, LIF, stand-off, explosives, fingerprints, scanning, laser spectroscopy, laser scattering, residue, remote

## Abstract

Here, we describe an innovative Integrated Laser Sensor (ILS) that combines four spectroscopic techniques and two vision systems into a unique, transportable device. The instrument performs Raman and Laser-Induced Fluorescence (LIF) spectroscopy excited at 355 nm and Laser-Induced Breakdown Spectroscopy (LIBS) excited at 1064 nm, and it also detects Laser Scattering (LS) from the target under illumination at 650 nm. The combination of these techniques supplies information about: material change from one scanning point to another, the presence of surface contaminants, the molecular and elemental composition of top target layers. Switching between the spectroscopic techniques and the laser wavelengths is fully automatic. The instrument is equipped with an autofocus and it performs scanning with a chosen grid density over an interactively-selected target area. Alternative to the spectroscopic measurements, it is possible to switch the instrument to a high magnification target viewing. The working distances tested until now are between 8.5 and 30 m. The instrument is self-powered and remotely controlled via wireless communication. The ILS has been fully developed at ENEA for security applications and it was successfully tested in two outdoor campaigns where an automatic recognition of areas containing explosives in traces had been implemented. The strategies for the identification of nitro-compounds placed on various substrates as fingerprints and the results obtained at a working distance of 10 m are discussed in the following.

## 1. Introduction

Remote measurements of the atomic and molecular composition of solid samples have a wide range of applications, among them are the analysis of pigments in cultural heritage, detection of explosives and their precursors, analysis of geological samples, measurements for forensics, space exploration, and industrial and environmental monitoring. The most-used techniques for remote molecular analysis of solids are Raman [1,2,3,4,5] and Laser-Induced Fluorescence (LIF) [6,7,8,9] while Laser-Induced Breakdown Spectroscopy (LIBS) supplies information about the elemental composition of the environment [10] or of the top target layers [11,12,13,14,15,16].

The three spectroscopic methods (Raman, LIF, and LIBS) provide complementary information and they might have some common components like a laser source, beam focusing, a signal collection system, as well as spectrometers. These facts have prompted an interest and several efforts to develop a sensing device that integrates LIBS and Raman [17,18,19,20] or all three techniques, as discussed in the following. Osticcioli et. al. [21] built a combined instrument for proxy (working distance of 2.5 cm) characterization of objects related to cultural heritage. They switched manually a single laser source between 532 nm for Raman and LIBS and 266 nm for LIF, then inserted or removed various filter and changed by PC commands the monochromator gratings to get High Resolution (HR) Raman spectra or lower resolution LIBS and LIF spectra in a larger spectral interval.

The first laboratory instrument for remote sensing by the three techniques was described in [22], aimed also at planetary exploration. This combined instrument contains a single laser source emitting at 532 nm and a single detector covering the spectral range of 535–680 nm. The Raman signal can be partially separated from LIF emission by restricting the acquisition gate in the proximity of the laser pulse arrival. The LIBS measurements with the described system suffer from a limited detected spectral range where the only lines observed from a rock material belong to Fe, Na, Ca, and Li.

The most advanced Raman-LIF-LIBS device reported until now regards the SuperCam instrument, intended to be mounted on a rover and used by NASA for the Mars 2020 mission [23,24]. The SuperCam integrates many remotely-controlled instruments, among them there is LIBS excited at 1064 nm plus Raman/LIF generated at 532 nm, with the measuring range up to 7 m and 12 m, respectively. The instrument is equipped with different spectrometer channels covering certain ranges in UV, VIS, and NIR, thus providing a large number of elements detectable by LIBS.

In the present paper we describe a patent-pending ENEA system ILS for remote material characterization by Raman, LIF, LIBS, and LS techniques. The instrument is equipped with two vision systems, it is fully automatized and has also a scanning capability. Although the ILS might have many applications, some of them were already tested like forensics, characterization of rocks or objects related to cultural heritage, etc., here we describe the system itself and show the preliminary results on explosives in traces, the detection of which still remains a challenging issue [2,3,4,5,7,15,18,25], particularly on remote targets [26]. The contaminants were left on substrates as fingerprints, which are considered as the most important mechanisms for the transfer of trace amounts of explosives during bomb handling and preparation [27].

## 2. Experimental

### 2.1. Overview

**Figure 1:**
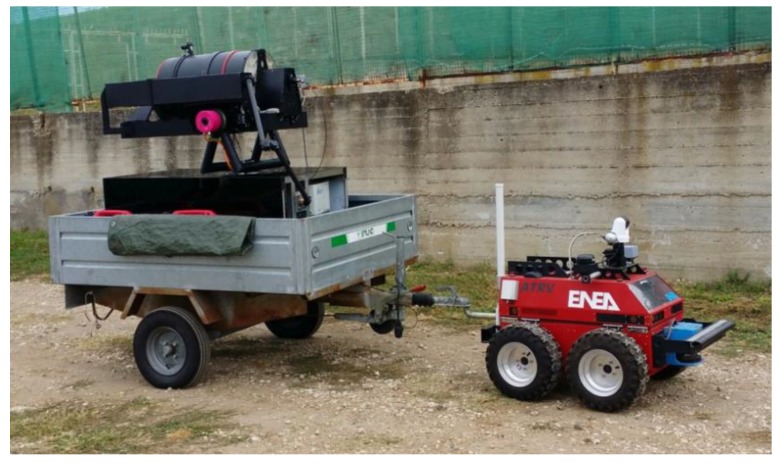
Integrated Laser Sensor (ILS) Prototype 2 placed on a trailer and driven by a remotely-operated vehicle.

**Figure 2:**
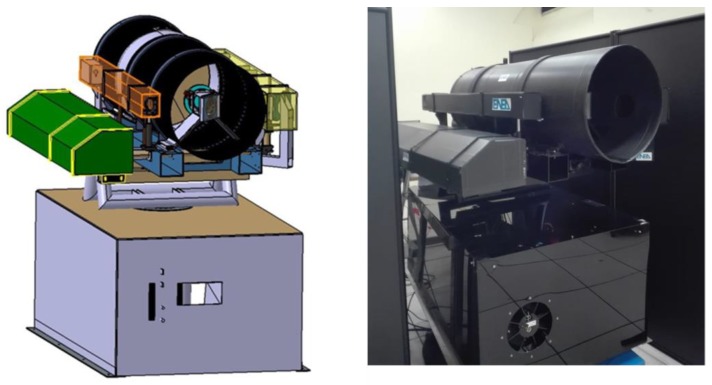
Drawing (**left**) and photo (**right**) of the ILS Prototype 3.

The ILS instrument was initially developed as a laboratory prototype with separate controls for each device and with manual movement of the optical components mounted on micrometric slits. After optimization of the optical lay-out, the components were integrated into a fully-automatized Prototype 2 (Figure 1), controlled remotely by home-developed software. This system had been successfully tested at the end of 2015 during one demo campaign. Next, we built Prototype 3, where the mechanical stability was improved by integrating the laser head into the rotating platform (Figure 2). In the following, we describe and discuss the last instrument version (Prototype 3).

The ILS consists of the instrument box and the platform rotating both horizontally and azimuthally. The rotating platform carries the optical head of a high-power laser, the diode laser for measuring the target distance, two video cameras, optical components for launching the laser beams, then the telescope for the signal collection, and behind it, the multiplexer box for signal delivery. The instrument box encloses two spectrometers, various power supplies, control electronics, the motors of the scanning system, an air-cooling system, a PC server, and a WiFi transmitter/receiver. The two units are connected electrically via an umbilical cable while the two optical fibers deliver the signal to the respective spectrometers. The overall system specifications are given in Table 1.

The LS measurements here are intrinsically eye-safe while LIF and Raman spectroscopy could be performed in eye-safe regime by limiting the laser energy and number of pulses. For example, according to the ANSI Standard, in eye-safe measurements with only one delivered ns laser pulse at 355 mm, the maximum permissible exposure is 5.6 mJ/cm^2^. 

### 2.2. Optical System

**Figure 3:**
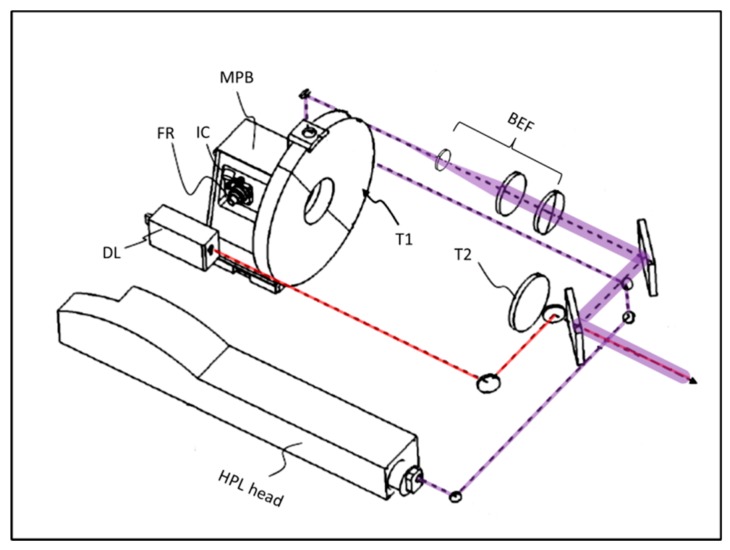
A simplified drawing of the overall optical system where DL is the distance-meter, T1 and T2 are telescope mirrors, MPB is the multiplexer box, FR is the fiber attachment for Raman, and IC is the internal camera. The violet thick line indicates the high-power laser beam, while the red line indicates the beam from the DL.

The optical system of the ILS is schematically drawn in Figure 3. The high-power laser (Q-smart 850, Quantel, Newbury, UK) emitted 6 ns-long pulses at a repetition rate up to 10 Hz and with a maximum energy of 850 mJ and 230 mJ at wavelengths of 1064 nm and 355 nm, respectively. Before and after the frequency conversion unit, there is a motorized slit carrying two laser mirrors each, used to bypass the nonlinear crystals and so delivering the beam at fundamental harmonics for LIBS measurements (not shown). Alternatively, the mirrors do not intercept the laser beam exiting the resonator, so the fundamental beam is converted to 355 nm and used for Raman and LIF spectroscopy. Both the UV and the IR beam are centered at the exit aperture of the laser head unit and aligned angularly to follow the same optical path. 

In order to reduce the length of the rotating unit, the high-power laser beam exiting the laser head is bent by mirrors (HR 1064/355) and brought to the back side of the platform. The laser radiation then passes through the beam expander/focuser, the last lens of which has a diameter of 100 mm. The first lens of this optical unit is mounted on a motorized slit in order to adjust the focus according to the target distance and the laser wavelength. The expanded high-power laser beam is injected into the telescope’s axis by means of two dielectrically-coated mirrors that are transparent to radiation of the laser diode (650 nm) emitted by the distance-meter (DIMETIX, FLS-C10 nm). The red beam is brought along the telescope’s axis by two metallic mirrors, and the second one is placed behind the last mirror for the high-power laser beam. 

The optical signal from the target is collected and focused by a Cassagrain telescope, the primary mirror of which has a diameter of 400 mm. Both telescope mirrors are coated with AlMgF in order to maximize the reflectivity in the UV spectral region. The primary mirror, together with the multiplexer box, is mounted on a motorized stage that keeps the target in focus when the working distance changed. The multiplexer box distributes the optical signal to one of the two optical fibers or to the internal camera, and it also inserts/removes the razor edge filter that cuts the UV laser radiation (not shown).

LIBS and LIF spectra are detected by the Echelle spectrometer (Mechelle 5000, Andor, Belfast, UK) equipped with an ICCD (iStar DH334T, Andor), covering the range of 200–900 nm. The quartz fiber connecting this unit with the multiplexer has an 800-µm core diameter. For collecting the intrinsically-weak Raman signal, we use a bundle containing 100-µm diameter fibers arranged at the exit into an array to match the slit of the Czerny–Turner spectrometer (iHR-320, HORIBA, Kyoto, Japan) equipped with a CCD detector (iKon-M934, Andor).

Due to the large achievable laser beam expansion (up to 10×) the minimum theoretical spot size on a target, calculated for the beam quality M^2^ = 2, is relatively small (Table 2). However, for LIBS, we consider the optimal spot size as a value corresponding to the maximum plasma emission that occurrs when focusing the beam beyond the target surface. In order to collect as much Raman signal as possible, for this technique, the high-power laser beam is defocused to match the diameter of the target area imaged by the telescope on the fiber bundle. For simplicity, the same laser spot size is used for LIF, although here and in the LS measurements, the signal is collected from a smaller area, corresponding to the image of the single, large core fiber. The lateral resolution of the measurements is given in Table 2, and it could be improved in Raman and LIF measurements by a tighter focusing of the high-power laser beam.

### 2.3. Software

**Figure 4:**
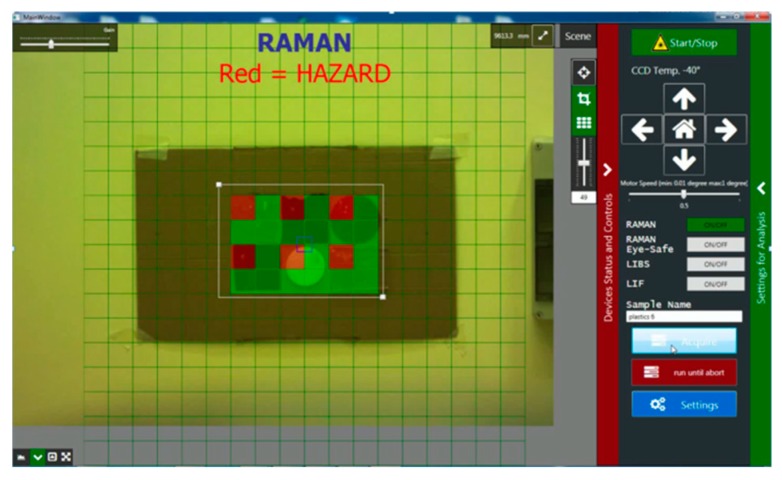
User interface photographed after Raman scanning of six plastic materials, each one containing an ammonium-nitrate fingerprint in the upper left corner. The largest area of the desktop is occupied by the image (video) acquired by the external camera. Fingerprints containing ammonium-nitrate were identified in real time, producing a red-colored cell in correspondence with its position on the target.

Software for control of the ILS instrument was developed in a Net.4 Windows environment. The program core activates and controls all the devices, which correct functioning allows launching the interactive interface to perform the spectroscopic measurements. The user’s interface on the desktop, photographed after target scanning by Raman technique, is shown in Figure 4. The central image is continuously taken by the external camera.

The signal processing routines, like background subtraction and de-noising of the Raman spectra, as well as peak identification to classify nitro-compounds starting from the built-in database, were written in LabView and recalled by the main program. The analogue routines were written for recognition of a generic nitro-compound by LIBS. For both techniques, the areas where a nitro-compound was detected appear in real-time as red cells on the target image. In the case of Raman measurements, the name of the identified nitro-compound and the corresponding measured spectrum appear on the desktop if clicking on the specific target’s point.

The internal camera is used only in specific cases, and it is presently controlled out of the main instrument’s program.

### 2.4. Materials and Methods

For the first outdoor campaign, the task was to identify fingerprints of nitro-compounds left on a vehicle. In preliminary laboratory measurements, we placed, with a silicon finger, residues up to the 10th generation on black polypropylene from the internal car and on white varnished metal from the external car. The analyzed residues included different military-grade explosives: trinitrotoluene (TNT), cyclotrimethylene-trinitramine (RDX), cyclotetramethylene-tetranitramine (HMX), and pentaerythritol-tetranitrate (PETN), plus their possible precursors as analytical-grade ammonium-nitrate and urea-nitrate, plus some common interferents like dust, wax, hand-cream, diesel, lubricating oil, and grease. Except for the LIBS, performed at 1–4 points per fingerprint, the measurements regarded the central part of fingerprint left on the substrate. Furthermore, inside the second outdoor campaign, we tested a limited number of fingerprints per substance on other substrates like textile, jeans, card, and some other plastic materials.

## 3. Results

In the multi-technique target scanning by the ILS, we usually apply the following sequence:

1. LIF and LS measurements for identifying eventual changes of the target material (roughness and surface contamination).
2. Eventual inspection of the previously-tagged interesting areas by the internal camera.
3. Raman measurements over the whole target or just over the previously-tagged areas, in order to obtain eventual molecular identification.
4. At the end, we performe LIBS measurements (micro-destructive) that supplies information about the elemental composition of the top sample layer(s).

### 3.1. LIF and LS Measurements

**Figure 5:**
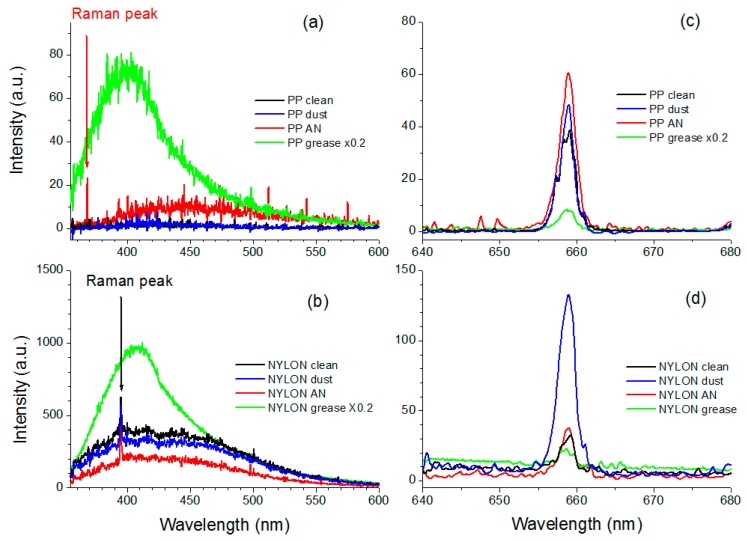
LIF spectra from polypropylene (PP) (**a**) and nylon (**b**) measured on clean area and on fingerprints containing ammonium-nitrate (AN), dust, or grease. The corresponding LS peaks are shown in the right panels (**c**,**d**).

The LIF measurements were performed at a target distance of 10 m by applying the laser energy of 16 mJ and the signal accumulation over 10 laser pulses. The acquisition was pre-triggered 500 ns before the high-power laser pulse, and the gate width was set to 0.1 ms as a compromise to capture both the expected short-living LIF signal and the scattered light under continuous-wave illumination by the red laser, without masking the signals by the ambient light. Examples of LIF + LS processed spectra (corrected for the spectral response and smoothed over 10 point) from black polypropylene and from white rough nylon containing clean areas or first-generation fingerprints of different contaminants, are shown in Figure 5. On the black polypropylene, we observed fluorescence from ammonium-nitrate, having a wide peak around 460 nm, together with its Raman peak (368.3 nm or 1044 cm^−1^). The grease residue had a very intense LIF signal peak around 420 nm. On the same black target we observed a wide LIF peak centered on 530 nm coming from all the examined nitro-compounds except for PETN. All the tested organic interferences had the LIF peak closer to the laser excitation wavelength (not shown). The detected LS signal increased in the presence of powder residues compared to the clean substrate while it dropped on a greasy area. We detected the differences in LS and LIF signals between the black clean substrate and all the examined fingerprints on the black substrate.

Differently, on the fluorescing nylon target also the Raman peak due to C-H bond (395.2 nm or 2900 cm^−1^) was observed and the examined powdered residues decreased the LIF signal intensity, i.e., they partially masked the substrate’s fluorescence. In this case as well, the greasy residues had an intense fluorescence, the peak of which was shifted towards the UV compared to the clean nylon surface. Again, compared to the clean area, the LS signal from any greasy print was low while it increased in the presence of a powdered material. Analogous results were obtained on the white varnished substrate, the fluorescence of which was higher than the characteristic one from the examined nitro-compounds placed as fingerprints.

### 3.2. Internal Camera

**Figure 6:**
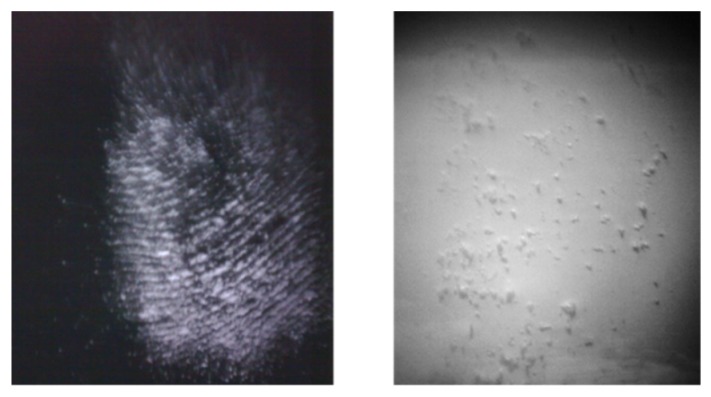
Traces of RDX left by a fingerprint and observed by the internal camera at a target distance of 10 m: **left**, on black polypropylene; **right**, on white varnished metal.

The internal camera captures the target image magnified through the telescope and its operation could be selected both for checking the surface state (Figure 6) and to point precisely the instrument. The latter capability was exploited to perform remote LIBS measurement exactly on the selected small features of the target [11].

### 3.3. Raman

**Figure 7:**
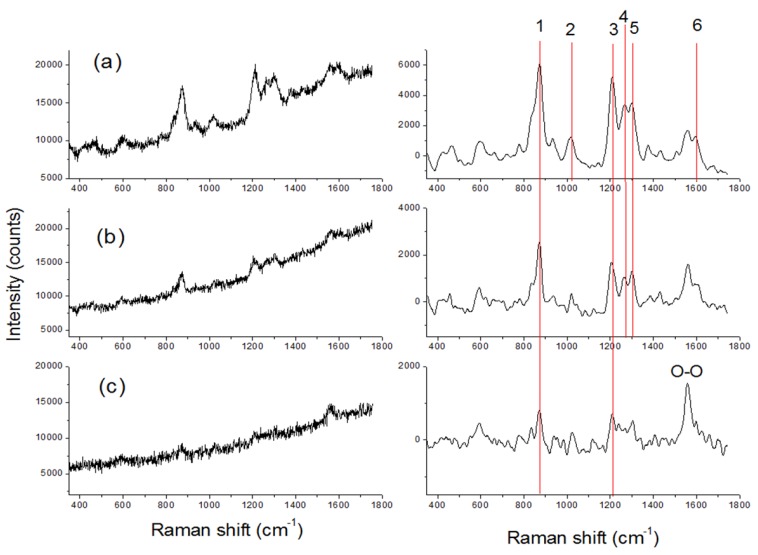
Raw (left) and processed (right) Raman spectra from RDX fingerprints of the 1st (**a**), 3rd (**b**), and 9th (**c**) generation placed on the white varnished metal. Red lines on the left panel indicate the RDX peaks detected automatically by the instrument’s software.

At a target distance of 10 m, by applying the laser energy of 16 mJ and the signal accumulation over 50 laser shots, we observed characteristic Raman peaks from the first 10 fingerprint generations containing any of the examined nitro-compounds except TNT. Figure 7, left, shows the raw Raman spectra detected from RDX fingerprints placed on the white varnished metal. The corresponding processed spectra and the RDX peaks detected automatically are depicted on the right panel. On the first-generation fingerprint, with the estimated RDX mass in the order of 1 mg, six characteristics peaks were detected (Figure 7d). On the third fingerprint, four peaks were assigned automatically (Figure 7e) while on the ninth-generation fingerprint only the two most intense Raman lines emerged from the noise. On the RDX fingerprint of the tenth generation (not shown) the only recognized peak was centered on 885 cm^−1^, corresponding to C–N–C symmetric ring breathing. In all the cases the vibration peak from O_2_ (1555 cm^−1^) was present in the spectra. The Raman peaks from RDX were not detected on fingerprints of the 4th, 6th, and 7th generations.

**Figure 8:**
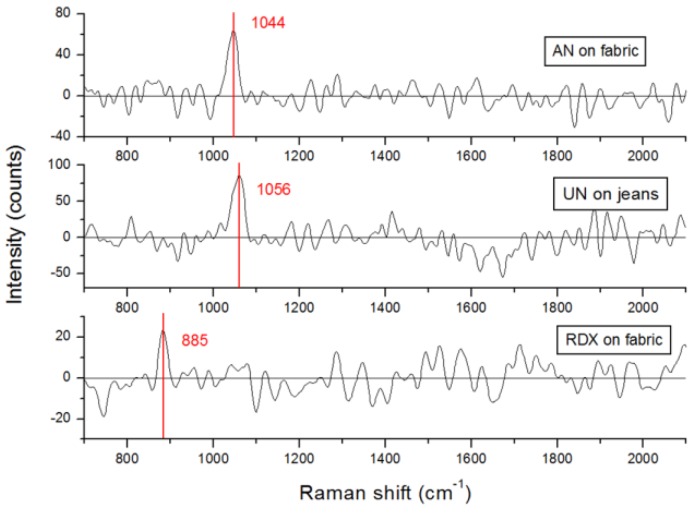
Processed single-shot Raman spectra in eye-safe regime measured on the first-generation fingerprints of ammonium-nitrate, RDX, or urea-nitrate placed on a dyed tissue at a 10 m distance from the instrument. Red lines indicate the peaks detected automatically by the instrument’s software.

In eye-safe regime we performed Raman measurements on different first-generation fingerprints at a distance of 10 m. Here, we applied a laser fluence of 4.6 mJ/cm^2^, slightly lower than the maximum permissible exposure, and a single shot acquisition. Successful Raman detection of ammonium-nitrate, urea-nitrate and RDX in the eye-safe regime was obtained also on fluorescing targets (Figure 8) while no peaks were observed from PETN and TNT.

**Figure 9:**
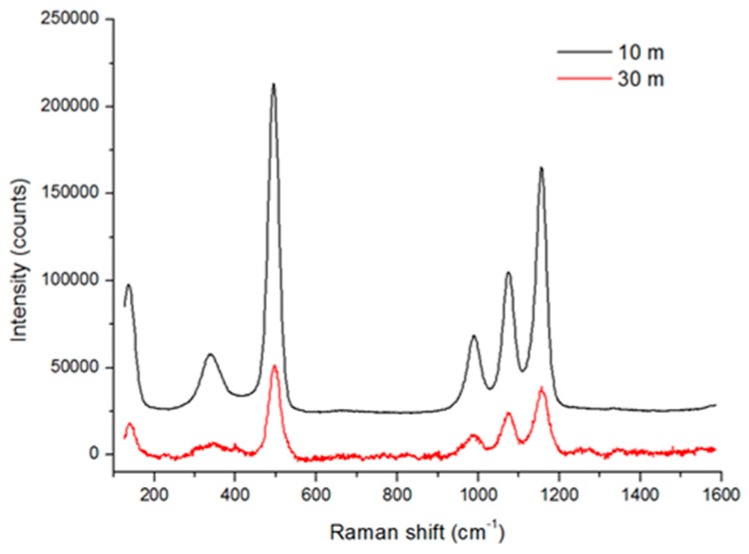
Raman spectrum from Teflon acquired at a target distance of 10 m (black line) and 30 m (red line) by accumulating the signal over 50 laser shots with the energy of 16 mJ.

Figure 9 compares the captured Raman spectra from Teflon placed at a distance of 10 m or 30 m for an incident laser energy of 16 mJ. At the higher distance the laser spot diameter, matching the area collected by the fiber bundle, was almost three-times larger (see Table 2). The corresponding peak intensities were about three-times weaker than at distance of 10 m. These differences could be attributed both to the smaller solid angle for signal collection and to larger radiation losses (scattering and absorption) when increasing the optical path through an air atmosphere. However, the Raman spectra here were acquired for the laser energy that was only 7% of the available while the energy density, also at a 10 m distance, was much lower than the threshold for damage of the target material. Consequently, we might conclude that Raman measurements on homogeneous materials could be performed with the same response when extending the detection range to 30 m and beyond if increasing the laser energy, here available up to 230 mJ.

### 3.4. LIBS

**Figure 10:**
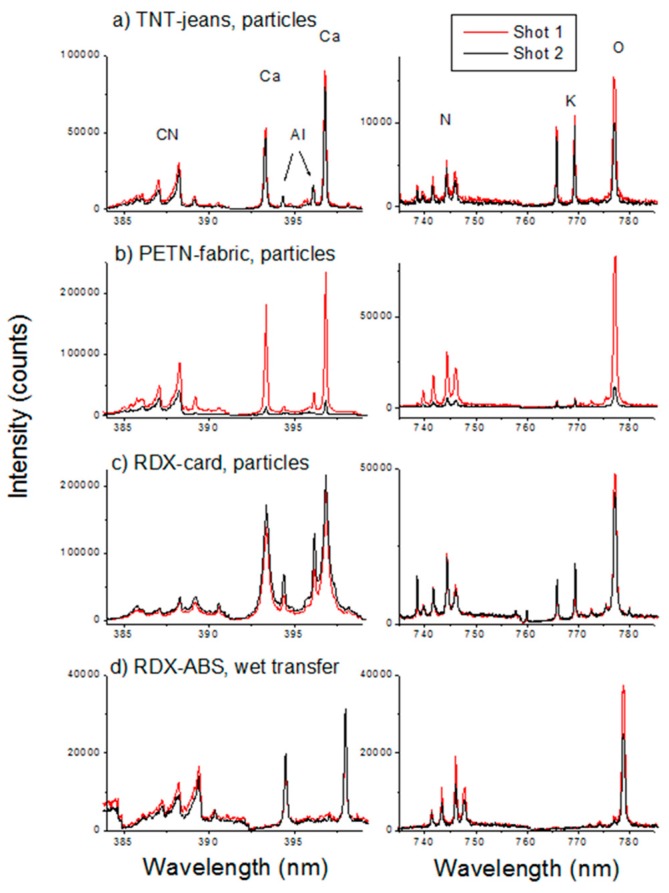
Single-shot LIBS spectra in intervals containing CN molecular features (left panel), N I and O I emission (right panel), for explosives in the form of particles (**a**–**c**) or delivered by a wet transfer (**d**) on different organic substrates.

At a working distance of 10 m the LIBS signal was created by pulses with the incident energy of 330 mJ. The spot size on the target was optimized for the maximum plasma emission intensity on the polypropilene sample, and it corresponded to a diameter of 1.3 mm. The spectra were acquired with the acquisition gate delay and a gate of 2.5 µs and 10 µs, respectively; the ICCD gain was set to 2500. At each sampling point three laser pulses were delivered, registering the spectrum after each shot. Particles around the sampled spot were blasted away by the laser-induced shockwaves, leaving an almost clean area of about 6 mm in diameter. For this reason, the LIBS sampling was performed at points distanced for at least 4 mm from each other. On different tested substrates, in the presence of nitro-compound particles the atomic line intensities from H, N, and O were higher after the first laser shot than after the second one (Figure 10, right panel) although the C I peak (not shown) and the UV CN lines (Figure 10, left panel) might have similar intensities in the both cases. This is particularly evident on substrates with a low ablation rate, as for example textiles (fabric, jeans). Differently, the areas contaminated by greasy residues showed a significantly reduced overall plasma intensity compared to the bare material. We exploited the differences in the spectra after the first laser shot, interacting with the surface residue, and the second shot, ablating the cleaned substrate, to achieve identification of the nitro-compounds also on organic substrates.

**Figure 11:**
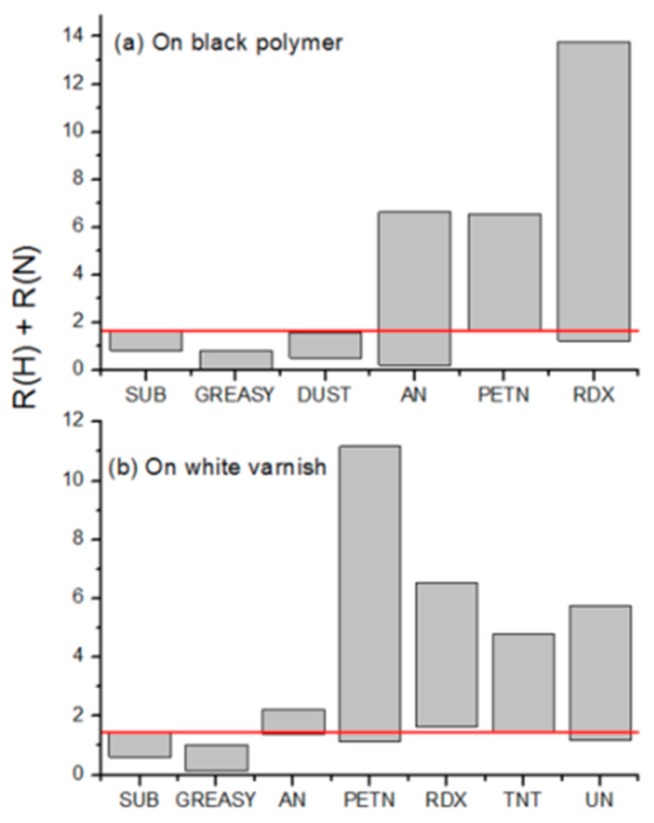
Span of the LIBS measured sum S of the ratios R(H) = I_1_ (H)/I_2_ (H) and R (N) = I_1_ (N)/I_2_ (N) on different fingerprints placed over black polypropylene (**a**) or white varnished metal (**b**). The red line indicates the lower threshold established for the detection of nitro-compounds. X axis: SUB is clean substrate, AN is ammonium-nitrate and UN is urea-nitrate.

Figure 11 shows the sum S of the ratio R (H) relative to Hα line peak after the first and the second laser shot and of the analogue ratio R (N) relative to the N I line (746.83 nm). We excluded the O I line from in the nitro-compound’s identification because some substrates might have surface oxidation, which could increase the intensity of this line after the first laser pulse compared to the second one. On the black polypropylene (Figure 11, top) the clean material and the same with dust particles had a very similar range of the sum S = R (H) + R (N) while the S value was significantly lower in presence of greasy residues. On the other hand, the sum S increases up to eight times when passing from the clean polypropylene to areas containing particles of ammonium-nitrate, PETN, or RDX (the only nitro-compounds tested on this substrate). Similar results were obtained on the white varnished metal where also TNT and urea-nitrate were tested.

In the ILS system, presently, the algorithm for the automatic recognition of nitro-compounds by LIBS is based on determining the sum S and assigning the hazard on the target’s cell if S > 1.6.

**Figure 12:**
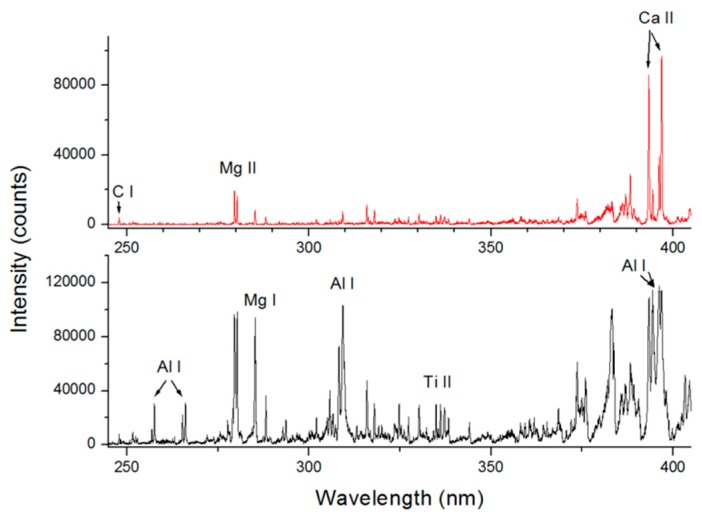
UV portion of a single-shot LIBS spectrum acquired at distance of 30 m on: top, Teflon substrate; bottom, aluminum target.

At a target distance of 30 m, the shortest wavelength of the lines detected by LIBS with the pulse energy of 330 mJ was around 227 nm, and this corresponded to Al I transitions from aluminum substrate. It was not possible to compare directly the LIBS spectra for distances of 30 m and 10 m because the optimized acquisition parameters were different in the two cases. However, at 30 m and in a single shot acquisition it was possible to observe, besides the lines from Al, also a number of impurities, as for example Mg, Ca, Ti, Si, Na, and K. The analogue probing of the Teflon sample (Figure 12, top) revealed the C I line at 247.8 nm and different impurities.

## 4. Discussion

It is well known that the number of particles and their distribution vary significantly from one fingerprint to successive prints [28,29], where the transfer efficiency also depends on the adhesive properties of the substrate [30]. For example, the first-generation fingerprint containing RDX particles might be heavily loaded, with a mass in the order of 1 mg, while already the fifth fingerprint contained a particle mass 1000-times lower [15]. The first prints rapidly loose the large-diameter heavy particles and then the transfer efficiency to the next prints, containing mainly small and light particles, is high. For example, in [29], it was found that the RDX mass measured for the fifth fingerprint was of 1.7 µg, while the 50th fingerprint had a mass of 0.23 µg. Furthermore, the prints did not exhibit a gradual progression from the heaviest loading to the lightest, and this might explain why we missed detecting RDX by Raman technique on some fingerprints, but recovered the signal at successive print generations.

To estimate roughly the detection sensitivity of the ILS techniques, we started from the typical RDX particle mass per unit area in fingerprints, based on the data reported in [29]. We encircled the area of the shown fingerprint on the dimensionally-calibrated photo of 29 × 18 mm and found that the print occupied about 4.1 cm^2^. The extrapolated particle mass of RDX inside the 10th fingerprint was about 1 µg, corresponding to the mean mass density of MD_10_ = 0.24 µg/cm^2^.

The LS-LIF signals at a distance of 10 m were collected from a spot diameter of 3.3 mm (area 0.085 cm^2^). On a non-fluorescing or weakly-fluorescing substrate, all the examined nitro-compounds excluding PETN showed their own LIF spectrum, detectable up to the 10th fingerprint, which corresponded to the mean probed particle mass of 20 ng. Furthermore, the LIF signal intensity might be improved by increasing the laser fluence inside the target area captured by the optical collection system. This could be achieved both by focusing the high-power laser beam more tightly down to a diameter of 3.3. mm or by increasing the laser energy from 16 mJ, used here, to, for example, 200 mJ. Raising the laser energy to 200 mJ in the same focusing conditions would lead to an energy density of 0.30 J/cm^2^, too low to induce ablation on most of the materials. In the case of RDX, the lower damage threshold under 355-nm irradiation by ns pulses is about 1.1 J/cm^2^ [31]. However, when working on the black polypropilene substrate we noticed some sound released during the beam-target interaction for laser energies above 60 mJ, caused by rapid surface heating although the substrate remained unaltered under visual inspection. Thus, in order to avoid thermal effects on any target, in the systematic measurements we limited the UV laser energy. From the previous considerations, we might conclude that for the fluorescing nitro-compounds on a non-fluorescing substrate placed at a 10-m distance, the particles might be detected by the LIF technique (of the ILS system) down to ~10 ng without altering the target. This sensitivity is sufficient to observe high-generation fingerprints although the LIF technique, itself, cannot be considered discriminatory for nitro-compounds. On the other hand, the examined liquid organic residues were highly fluorescing, but with the emission peak close to the laser excitation wavelength, and they might be detected in much smaller quantities than the nitro-compounds.

The detection sensitivity analogue to the LIF might be deduced also for the LS measurements on dark substrates. Here, the back-scattered signal from the continuous-wave red laser could be increased by applying longer acquisition times. While surface particles increased the LS signal compared to the dark substrate, the presence of greasy residues heavily lowered the backscattered signal, if present on the bare substrate. In this way, the combination of LS-LIF measurements was extremely useful for tagging the target areas containing some surface contaminants, supplying also information about the residue state: particles or liquid/gel.

In the case of a highly fluorescing and scattering substrates like the examined white varnished metal, the presence of residues might be deduced from changes in the LS-LIF spectral intensity and distribution (for LIF). In such a case, the sensitivity, both of the LIF and the LS measurements, is more difficult to estimate because the detection of residues is based on differences in the response between the bare and the contaminated area, and not on the absolute signal intensities.

Regarding the Raman measurements, the considerations were analogous to the LIF technique, but in this case the signal was collected from a larger area, corresponding to 0.66 cm^2^. Assuming the presence of 1 µg of a nitro-compound in the 10th-generation fingerprints, the Raman signals of which were weak but generally observable (excluding TNT), the instrument’s detection sensitivity at a 10-m distance was of about 150 ng. This detection limit could be further improved by increasing the energy of the high-power laser a few times in the absence of an intense substrate fluorescence that could mask the weak Raman signal.

In the LIBS measurements at a distance of 10 m, the energy density on the target was 24.9 J/cm^2^. This laser fluence was sufficient to create plasma on all the tested samples and it was well above the 100% ignition threshold for RDX [31] and PETN [32] under the analogue laser excitation. On residues of these secondary explosives the measured sum S achieved values > 11, more than six-times higher than those measured on the bare substrate. A few random missing detection points (S < 1.6) for RDX on the black polypropylene and for PETN on the white varnish could be attributed to a locally low particle presence inside the small laser spot. TNT was tested only on the white varnish and it was always detected by LIBS, although with S values < 5. Given that this residue was not observed by Raman, we believe that its presence on the target was lower than expected because of its relatively easy evaporation. 

Ammonium-nitrate and urea-nitrate, which are not explosives but oxidizers, are less sensitive to shock than the explosives. For these nitrates the determined S values were always <7. In particular, very low S values in some prints of ammonium-nitrate compared to the bare black substrate might be attribute to the hygroscopic properties of this substance, where the presence of the absorbed water highly reduced the plasma emission intensity. Beside the very low plasma emission intensity when the substrates were covered by liquid/gel residues, it is interesting to note that in the presence of dust contamination, the S values were equal to or smaller than on the clean substrate. This indicates that the increased LIBS intensities of the H I and N I (the elements also present in air) lines on areas containing explosives or oxidizers were not caused by an improved laser–particle interaction compared to the laser-substrate, but to the plasma temperature increase due to the laser-induced micro-explosions and endothermic reactions [31,32,33]

Considering that the laser spot in the LIBS measurements was of only 1.3 mm in diameter and assuming an uniform particle distribution, the 10th-generation fingerprint would contain about 3 ng of particles inside the probed spot. This mass could be approximately considered as the detection limit reached on the tested samples. In the future, the effect of higher laser energy density on the detection sensitivity by LIBS could be examined, but taking into account that a higher incident energy might increase the contribution of the target to the plasma produced on a surface residue.

The herein applied approach for the detection of nitro-compounds by LIBS, based on the spectral differences after the first and the second laser pulse at the same position, was much simpler and straightforward than the methods proposed in [15,33,34,35,36]. Our approach was not affected by missing plasma stoichiometry [36] and it reduced the influence of substrate material on the classification results. Furthermore, this method exploited only the emission lines in the red-NIR spectral region, which are less sensitive to the atmospheric attenuation than UV spectral features, and this is a promising factor for the stand-off LIBS detection of trace explosives at longer distances than reported in this work.

## 5. Conclusions

The herein described ILS system allowed scanning of remote targets, producing 2D thematic maps. By simultaneous LS and LIF and measurements it was possible to observe changes of the material from one point to another, to identify areas with the presence of surface residues and to have indications if the surface contaminants were liquid or in the form of particles. Visualization of the surface details was also possible by the internal camera, which works with a high magnification optical system. Raman probing allowed recognizing the molecular composition of residues (if present in a sufficient quantity) and tagging the areas where nitro-compounds were identified. Similarly, target scanning by LIBS tagged areas containing a generic nitro-compound. The estimated detection sensitivities for nitro-compound particles for a target distance of 10 m were in the order of 10 ng, 100 ng, and 1 ng for LS-LIF, Raman, and LIBS, respectively.

The ILS instrument is fully automatized, equipped with a user-friendly software interface controlled via wireless communication. These characteristics permits operating also in hazardous conditions, either from a fixed position or mounting the system on a robotic vehicle. Except for LIBS, the measurements could be performed also in eye-safe regime, but with reduced detection sensitivity. The tests were performed up to a distance of 30 m and using less than 10% or 40% of the available laser energy at 355 nm and 1064 nm, respectively. Consequently, it could be expected that the instrument’s maximum working distance might largely exceed 50 m.

In this work we focused on the instrument performances and its ability to detect specifically the nitro-compounds. Nevertheless, by recalling different data processing algorithms, the ILS could produce many other thematic maps, both molecular and elemental, among them for example: recognizing the polymer type or producing a distribution of heavy elements inside the scanned area. 

The ILS is a highly-performing scientific instrument and it is intended as a starting point for the development of other simplified instruments for some specific applications, such as characterization of cultural heritage objects, forensic, environmental monitoring and many others.

## 6. Patents

V. Lazic, A. Palucci, L. De Dominicis, M. Nuvoli, M. Pistilli, I. Menicucci, F. Colao, S. Almaviva, Dispositivo ILS (Integrated Laser Sensor) per le analisi di materiali con tecniche Raman, LIF (Laser Induced Fluorescence) e LIBS (Laser Induced Breakdown Spectroscopy). Patent deposited 28 December 2017 at Ministero dello Sviluppo Economico, Italy, Number 102017000150309.

## Figures and Tables

**Table 1:** Specification of the ILS system.

Feature	Specifications	Notes
Distance measurements	Limit 500 m ^(1)^, resolution 0.1 mm	^(1)^ On reflective target
Target distance	8.5–30 m ^(2)^	^(2)^ tested; estimated >100 m
Autofocus	Yes	
Scanning resolution	0.1 mrad	1 mm at a distance of 10 m
LIF measurements	Laser @355 nm, filter ^(3)^, Echelle spectrometer	^(3)^ Razor edge 355 nm
Scattering measurements	Laser @650 nm, filter ^(3)^, Echelle spectrometer	Simultaneous with LIF
Raman measurements	Laser @355 nm, filter ^(3)^, Czerny-Turner ^(4)^	^(4)^ Higher throughput than Echelle
LIBS measurements	Laser @1064 nm, Echelle spectrometer	
Switching time between two measuring techniques	10 s ^(5)^	^(5)^ Limited by the software
External camera	Color, 1280 × 1024 pixel, with objective f 35	Always acquiring
Internal camera	Color, 1280 × 1024 pixel, magnification 3.5X − 22 × for the target distance of 8.5−30 m	Alternative to the spectroscopic measurements
Control	WiFi and the dedicated software ^(6)^	^(6)^ ENEA’s property
Ambient temperature	10 °C ^(7)^–35 °C	^(7)^ tested 10 °C–40 °C

**Table 2:** The minimum theoretical laser spot size for three target distances, the used laser spot size, and the lateral resolution for Raman, LIF and LIBS measurements.

Distance (m)	Th. Spot ø (mm) 355/1064 nm	Used Spot ø (mm) 355/1064 nm	Area ø (mm) Imaged on the Fiber Bundle	Area ø (mm) Imaged on the Large Core Fiber	Lateral Resolution (mm) Raman/LIF/LIBS
8.5	0.19/0.55	7.2/1.1	7.7	2.8	7.2/2.8/1.1
10	0.22/0.68	9.2/1.3	9.2	3.3	9.2/3.3/1.3
30	0.67/1.96	28.2/2.2	28.6	10.4	28.6/10.4/2.2
